# Do Candidate Genes Mediating Conspecific Sperm Precedence Affect Sperm Competitive Ability Within Species? A Test Case in *Drosophila*

**DOI:** 10.1534/g3.114.012476

**Published:** 2014-07-16

**Authors:** Alberto Civetta, Scott Finn

**Affiliations:** Department of Biology, University of Winnipeg, Winnipeg, MB, R3B 2E9 Canada

**Keywords:** **s**perm competition, conspecific sperm precedence, gene co-option, pleiotropy

## Abstract

When females mate to multiple males, the last male to mate fathers the majority of progeny. When males of different species inseminate a female, the sperm of the male conspecific to the female is favored in fertilization in a process known as conspecific sperm precedence (CSP). A large number of studies in *Drosophila* have assayed the genetic basis of sperm competition, with a main focus on *D. melanogaster* and accessory gland protein genes. Only a few studies have attempted to disentangle the genetic basis of CSP between related species of *Drosophila*. Although there is no *a priori* reason to believe that genes influencing intraspecific sperm competitive ability might also mediate conspecific sperm precedence, no study has addressed the question. Here, we test a group of candidate CSP genes between *D. simulans* and *D. mauritiana* for their effect on sperm competition in *D. melanogaster*. The use of *P*-element insertion lines identified CG14891 gene disruption as the only one causing a significant decrease in second male paternity success relative to wild-type and ebony tester males. The gene disruption affected both sperm displacement and the sperm fertilizing ability. Out of five genes tested using RNA interference, only gene knockdown of CG6864 (*Mst89B*) significantly reduced the male’s ability to father progeny when second to mate. Our results suggest that CG14891 and CG6864 might have been co-opted from an intraspecies gene function (*i.e.*, sperm competition) into an interspecies avoidance phenotype (*i.e.*, CSP). Alternatively, the dual role of these genes could be a consequence of their pleiotropic roles.

The phenomenon of sperm competition in *Drosophila melanogaster* has been extensively studied using mating laboratory assays as well as surveys of parenthood from wild-caught females. Overall, studies show that when *Drosophila* females mate with two or more males, the last male to mate has the greatest paternity success (Clark 2002). Second male paternity advantage holds also in heterotypic crosses involving *D. melanogaster* cosmopolitan and Zimbabwe races (Dixon *et al.* 2003).

Several genes have been tested for their role in competitive success during multiple mating using both gene manipulation assays and tests that associate gene sequence polymorphism with variation in sperm competitive success measured from paternity scores. The focus has been mainly on male accessory gland proteins (*Acp*s). For example, the deletion of *Acp62F* increases the male’s ability to place sperm in storage, and significant associations have been found between polymorphisms at *Acp62F* and sperm competitive ability (Fiumera *et al.* 2007; Mueller *et al.* 2008). *Acp29AB* and *Acp36DE* are needed for sperm storage and they also show significant associations between sequence polymorphism and variation in sperm competitive ability (Clark *et al.* 1995; Fiumera *et al.* 2005; Wong *et al.* 2008; Avila and Wolfner 2009). Other *Acps* have been found to be required for the proper utilization of stored sperm for fertilization (Avila *et al.* 2011). There is also both indirect and direct evidence of non-Acp genes influencing sperm competitive ability. Associations have been found between four genes within the X chromosome, a chromosome with a scarcity of genes for seminal proteins, and differences in sperm competitive ability in *D. melanogaster* (Greenspan and Clark 2011). Recently, deletion of the X-linked genes of the sperm dynein intermediate chain (*Sdic*) multigene family in *D. melanogaster* has been shown to affect sperm competitive ability without disrupting sperm motility or male fertility (Yeh *et al.* 2012).

We have recently gained a large amount of knowledge regarding the mechanisms of sperm utilization and competition by using males with fluorescently labeled sperm. For example, the use of transgenic males of *D. melanogaster* with fluorescently labeled sperm showed an important role played by second male displacement of resident sperm driven by sperm numbers (Civetta 1999; Manier *et al.* 2010). More recent studies have uncovered a common set of sperm precedence mechanisms shared among closely related species of *Drosophila*, yet a complex scenario of rapid diversification. For example, male sperm characteristics such as differences in sperm length can contribute to sperm advantage, but females can influence sperm utilization through ejection or by controlling the use of sperm for fertilization (Lupold *et al.* 2013; Manier *et al.* 2013a,b,c).

The observation of conspecific over heterospecific male advantage to father progeny when in competition has proven to act as a postmating prezygotic reproductive isolation barrier between species of *Drosophila* as well as other insects (Price 1997; Howard *et al.* 1998; Rugman-Jones and Eady 2007; Matsubayashi and Katakura 2009; Larson *et al.* 2012; Tyler *et al.* 2013). Among species of the *Drosophila simulans* clade, double mating of females to conspecific and heterospecific males have shown that the majority of progeny is fathered by the conspecific male regardless of the mating order, with the breakdown in competitive success of the heterospecific males being attributed to poor sperm displacement ability (Price *et al.* 2000, 2001). More recently, sperm dumping by females, or simply poor sperm storage, has been reported to influence the fate of heterospecific sperm in other species of *Drosophila* as females rapidly lose sperm from males of related species (Matute and Coyne 2010; Sagga and Civetta 2011). A recent study directly addressed the occurrence and causes of CSP between *D. simulans* and *D. mauritiana* by competing males with differentially labeled sperm. Despite differences between species, the study revealed many commonalities between previously known intraspecific mechanisms of sperm precedence and CSP, including a major role of sperm displacement and female control of sperm utilization through sperm ejection and fertilization biases (Manier *et al.* 2013c).

Only a few studies have attempted to map the genetics of CSP in *Drosophila* and other insects (Civetta *et al.* 2002; Britch *et al.* 2007; Levesque *et al.* 2010). The lack of attempts has probably been a consequence of not having a clear understanding of mechanisms; however, it is feasible to map loci that contribute to the phenotypic manifestation of CSP, *i.e.*, conspecific advantage to father progeny. We have used *D. simulans* lines with mapped genetic introgressions from *D. mauritiana* to test the effect of different heterospecific third chromosome introgressions on *D. simulans* second male paternity success. We identified a group of candidate CSP genes based on mapped loci with combined analysis of adaptive gene coding sequence diversification and male reproductive gene expression (Levesque *et al.* 2010). Given the commonalities in mechanisms of sperm utilization and precedence between species, we used a combination of *D. melanogaster P*-element insertions and RNAi constructs to test the effects of 10 previously identified CSP candidate genes on second male paternity success in *D. melanogaster*. *P*-element insertions were used to disrupt genes that have previously shown an effect on CSP based on coding sequence diversification, whereas RNAi knockdowns were used to target CSP candidate genes on the basis of differential gene expression. We found evidence in support of two CSP genes, CG14891 and CG6864, as influencing sperm competition within *D. melanogaster*. The results indicate the possibility of co-option of gene function from intraspecific male fitness to a role in interspecies hybridization avoidance. It is alternatively possible that both genes might have come to influence both intraspecies and interspecies phenotypes as a consequence of gene pleiotropic effects.

## Materials and Methods

### Phenotypic test of candidate genes in *D. melanogaster*

Drosophila stocks carrying *P*-element insertions at genes of interest were obtained from the Bloomington or Exelixis stock centers. Transgenics carrying an inducible UAS-RNAi construct for genes of interest were purchased from the Vienna Drosophila RNAi Center (VDRC) and a Dicer (Dcr) GAL4 driver stock (25708) from the Bloomington Drosophila Stock Center (BDSC) (Table 1). Flies were reared in cylindrical polypropylene bottles containing cornmeal–molasses–yeast–agar (CMYA) medium at 24°C in a 12-hr light–dark cycle. For controlled crosses, virgin female flies were collected and maintained in polypropylene vials containing fresh CMYA medium, with no more than 20 copies per vial.

**Table 1:** List of *Drosophila melanogaster* strains used

Strain	Source	Genotype	Construct	Gene affected
6550	Ward	Wild-type	NA	NA
1658	BDSC	e/e	NA	ebony
18320	BDSC	+/TM6Be, Tb	PBac	CG7478(Actin 79B)
18237	BDSC	+/TM6Be, Tb	PBac	CG31542
18348	BDSC	TM6Be, Tb	PBac	CG7362
24853	BDSC	e/TM3e, Sb	P	CG3158(*spindle-E*)
26029	BDSC	Wild-type	Mi	CG14891
F01790	Exelixis	Wild-type	PBac	CG6255
19276	BDSC	+/TM6Be, Tb	P	CG31287
102118	VDRC	Wild-type	RNAi	CG14891
102666	VDRC	Wild-type	RNAi	CG6864 (Mst89B)
103437	VDRC	Wild-type	RNAi	CG31287
40461	VDRC	Wild-type	RNAi	CG3610
109692	VDRC	Wild-type	RNAi	CG4836
25708	BDSC	UAS-Dcr2; Act5C-GAL4/ CyO	Driver	NA

BDSC, Bloomington Drosophila Stock Center; VDRC, Vienna Drosophila RNAi Center. Transposable element inserts are: PBac, PiggyBac; *P*, P-element; Mi, Minos; NA, not applicable.

**Figure 1:**
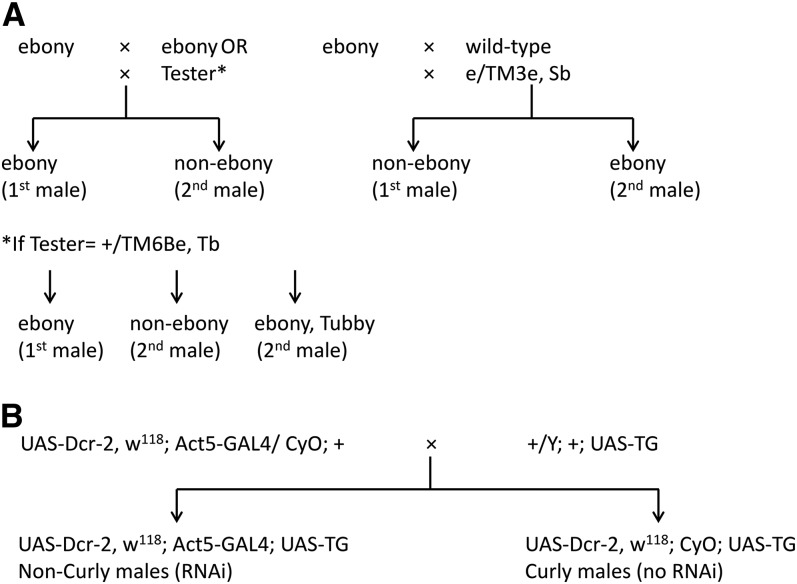
Schematic diagram of design for sperm competition assays (A) and creation of tester males for gene knock down assays (B). TG, target gene.

Males from lines with *P*-element gene insertions were used as tester second males to mate to females homozygous for an ebony (*e*/*e*) mutation that had already been singly mated to same-aged ebony males (male 1). Similarly, F1 males from the cross between the UAS-RNAi construct stocks and the Dcr-GAL4 driver stock (25708) were used as second tester males (Figure 1). All matings were observed every 15 min for a total period of 8 hr to avoid multiple mating to any one male. After the first mating, females were stored in a vial for 2 d (vial 1), at which point they were paired for a second mating with the tester males (male 2). Four days after the second mating, females were transferred to a fresh vial (vial 3). Progeny from vials 1, 2, and 3 were counted on the 20^th^ day after the beginning of oviposition and scored based on phenotypic body coloration, with ebony progeny being fathered by the first male and nonebony progeny being fathered by the second male. Some gene inserts were over balancers that segregate ebony (Table 1), scores were corrected for segregation bias by monitoring the Tubby phenotype (*e.g.*, +/e and e/e *vs.* e/e, Tb). For stock 24853, which is phenotypically ebony (Table 1), males from a wild-type stock (6550 from Ward’s Natural Science) were used as first to mate. We also tested males from the wild-type and the ebony (1658 BDSC) stocks as second to mate. Females that did not produce progeny from male 1 in vial 1 were discarded from further analysis (*i.e.*, no first mating). The fraction of progeny in vial 2 and vial 3 sired by the tester male was designated as P2.

We corrected P2 scores for differences in egg-to-adult viability of competing developing genotypes in a vial (Gilchrist and Partridge 1997). Viability was scored by crossing ebony females to tested males of wild-type phenotype or the +/TM6Be, Tb stock (Table 1). In tests involving wild-type males, the F1 females were backcrossed to ebony color males, and deviations from 1:1 segregation of wild-type to ebony body color phenotypes were scored. For the test crosses with +/TM6Be Tb males, e/+ F1 females were crossed to e/TM6Be Tb F1 males, and the segregation of wild-type, ebony color, and tubby size with ebony color provided counts to correct viabilities. Finally, for the tester males from stock 24853 (e/TM3e, Sb) (Table 1), ebony females were crossed to wild-type males (stock 6550 from Ward’s Natural Science) and F1 females (e/+) were crossed to males from stock 24853. The segregation of wild-type to ebony color gave the counts used for corrections. The viability correction factor is described elsewhere (Clark *et al.* 1999), but it briefly involves dividing scores in the sperm competition trials by twice the ratio of wild-type offspring sired by females in the noncompetitive backcross mating assays described above.

### Gene expression data analysis

The effectiveness of the Dcr-GAL4 driver to trigger downregulation of the responder gene construct via RNAi induction (Figure 1) was measured using quantitative reverse-transcriptase PCR (qRT-PCR). We collected and aged males for 4–6 days and dissected single abdomens from each line. Single fly abdomen samples were stored in lysis buffer at −70°C until RNA extraction. RNA was extracted using the Qiagen RNeasy plus mini kit following the manufacturer protocol. cDNA was synthesized using an iScript select cDNA synthesis kit (BioRad) and cDNA was quantified using a nanophotometer so that equal amounts of total cDNA were used in each qRT-PCR reaction. qRT-PCR was performed using the IQ SYBR Green Supermix kit from BioRad and performed in a MiniOpticon PCR System (BioRad). We ran PCR reactions from at least three biological replicates (different RNA samples) for each *Drosophila* line tested.

Primers for qRT-PCR were designed using Primer3 (http://frodo.wi.mit.edu/) to span an exon/intron boundary when possible, because it helped further control for possible DNA contamination. The efficiency of all primers pairs was tested by creating a standard curve using the threshold cycles generated from a dilution series of a template (amplified by qRT-PCR) and plotting those threshold cycle values against the log of the template dilution used in each reaction. The slope of the standard curve was used to assess primer efficiency (Supporting Information, Table S1). Rpl32 was used to calibrate expression, eliminating error from differences in RNA concentration between samples. After amplification, we tested for the presence of single amplification products by using a melting step using 0.5°C increments between 55° and 99°C and 1-sec hold reads. The ΔCT of the tested sample was calculated by subtracting the CT of the reference gene (RpL32) from the CT of the target gene. For each gene, relative expression of the knockdown was normalized by setting the average ΔCT of the F1 Curly males (no knockdown) (Figure 1) as calibrator.

### Data analysis

Phenotypic data were analyzed using one-way ANOVA with strains as the main factor followed by *a posteriori* Scheffe’s test. Pair-wise comparisons regarding the effect of gene knockdowns on both gene expression and phenotypic scores were performed using two-sample *t*-test.

## Results

### Comparison of second male paternity success among transposable element insert lines

**Figure 2:**
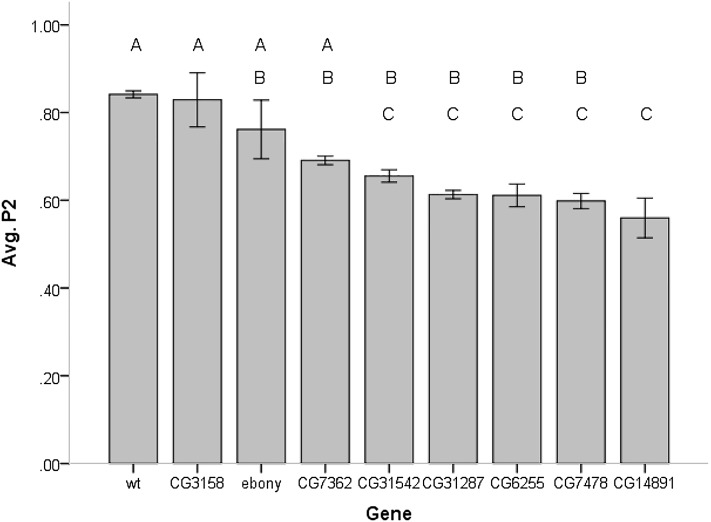
Average second male paternity of males carrying P-element insertions at different candidate genes. Error bars represent ± 1 SEM. Shared letters above columns indicate that the averages are not statistically different (*post hoc* Scheffe’s test). wt, wild-type.

**Figure 3:**
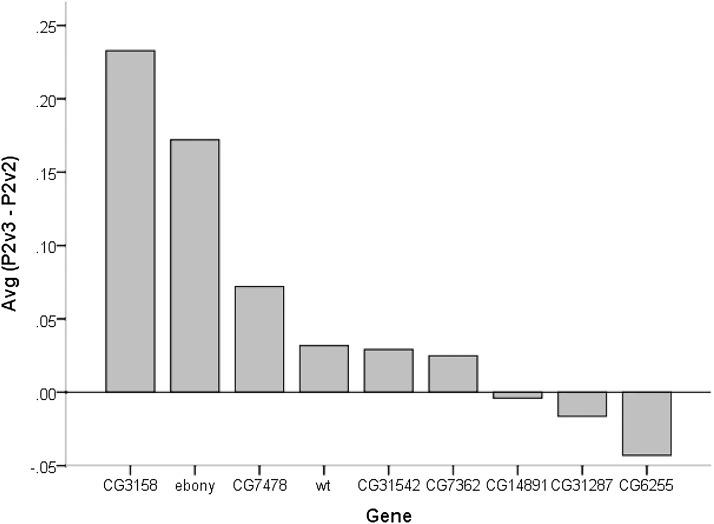
Average of the difference between second male paternity in vial 3 (P2v3) and in vial 2 (P2v2) for males carrying P-element insertions at different candidate genes.

We tested second male paternity success for seven strains of *D. melanogaster* carrying transposable elements inserts for genes of interest, as well as an ebony mutant and a wild-type strain (File S1). The setting involved scoring phenotypes for 20,411 progeny produced by 290 females. We found slight viability deviations (Table 2) and P2 values were corrected for differences in viability of the genotypes being scored (see *Materials and Methods*). We detected significant differences among strains in second male paternity success (F_8, 230_= 15.86; *P* < 0.001). Not surprisingly, the average sperm competitive ability of males from the different strain tested was correlated with the fecundity of the average male (r = 0.14; *P* = 0.026). Males from five strains corresponding to transposable elements inserts affecting CG14891, CG7478, CG6255, CG31287, and CG31542 had the lowest average P2 scores, ranging from 0.56 to 0.66, and significantly lower than wild-type males. However, only the strain with an insert within CG14891 had an average P2 score lower than both wild-type and ebony males (Figure 2). An examination of the immediate effect (vial 2) of gene disruptions on second male mating success showed significant differences among strains in P2 (F_8, 229_= 9.41; *P* < 0.001) with CG14891 (P2= 0.52), CG7478 (P2= 0.56), and CG6255 (P2= 0.60) having average P2 scores significantly lower than wild-type but not ebony tester males. The delay effect of gene disruption on second male paternity success (vial 3) also showed significant differences among treatments (F_8, 228_= 22.48; *P* < 0.001) with CG14891 (P2= 0.52), CG6255 (P2= 0.56), CG31287 (P2= 0.60), and CG7478 (P2= 0.63) gene inserts having significantly lower average P2 scores than both wild-type and ebony flies. Three strains carrying gene disruptions of CG14891, CG31287, and CG6255 show no increments in average second male paternity success from the second to third vial (Figure 3). Overall, only gene disruption of CG14891 had an effective and significant consequence in decreasing the overall competitive ability of males when second to mate, with disruption of other genes having only temporary effects.

**Table 2:** Viability correction factors (prop wt)

P-strain	Gene	N	Prop wt	RNAi strain	Gene	N (Cy; +)	Prop wt (Cy; +)
18237	CG31542	23	0.53	102118	CG14891	14; 34	0.59; 0.56
18320	CG7478	18	0.54	103437	CG31287	19; 33	0.58; 0.54
18348	CG7362	22	0.53	102666	CG6864	10; 24	0.55; 0.55
24853	CG3158	10	0.50	40461	CG3610	10; 10	0.53; 0.55
26029	CG14891	12	0.51	109692	CG4836	21; 10	0.51; 0.51
F01790	CG6255	20	0.55				
19276	CG31287	22	0.60				

N= Number of females assayed.

### Comparison of gene knockdown effects on second male paternity success

**Figure 4:**
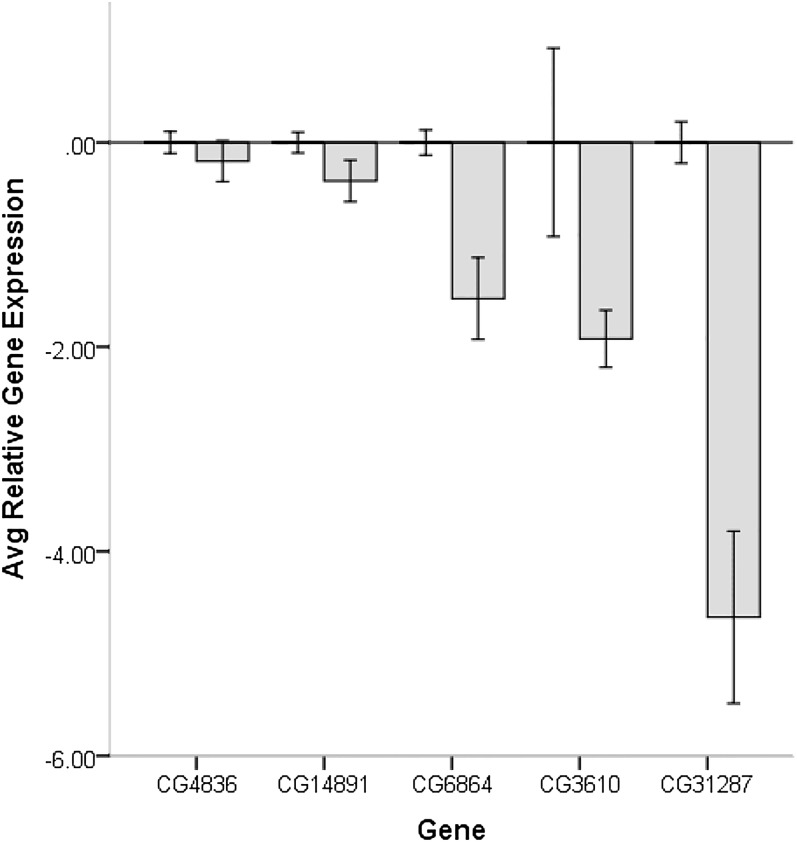
Average relative gene expression of males with a knockdown of candidate genes (light gray bars) relative to controls (average at zero). Error bars represent ± 1 SEM.

**Figure 5:**
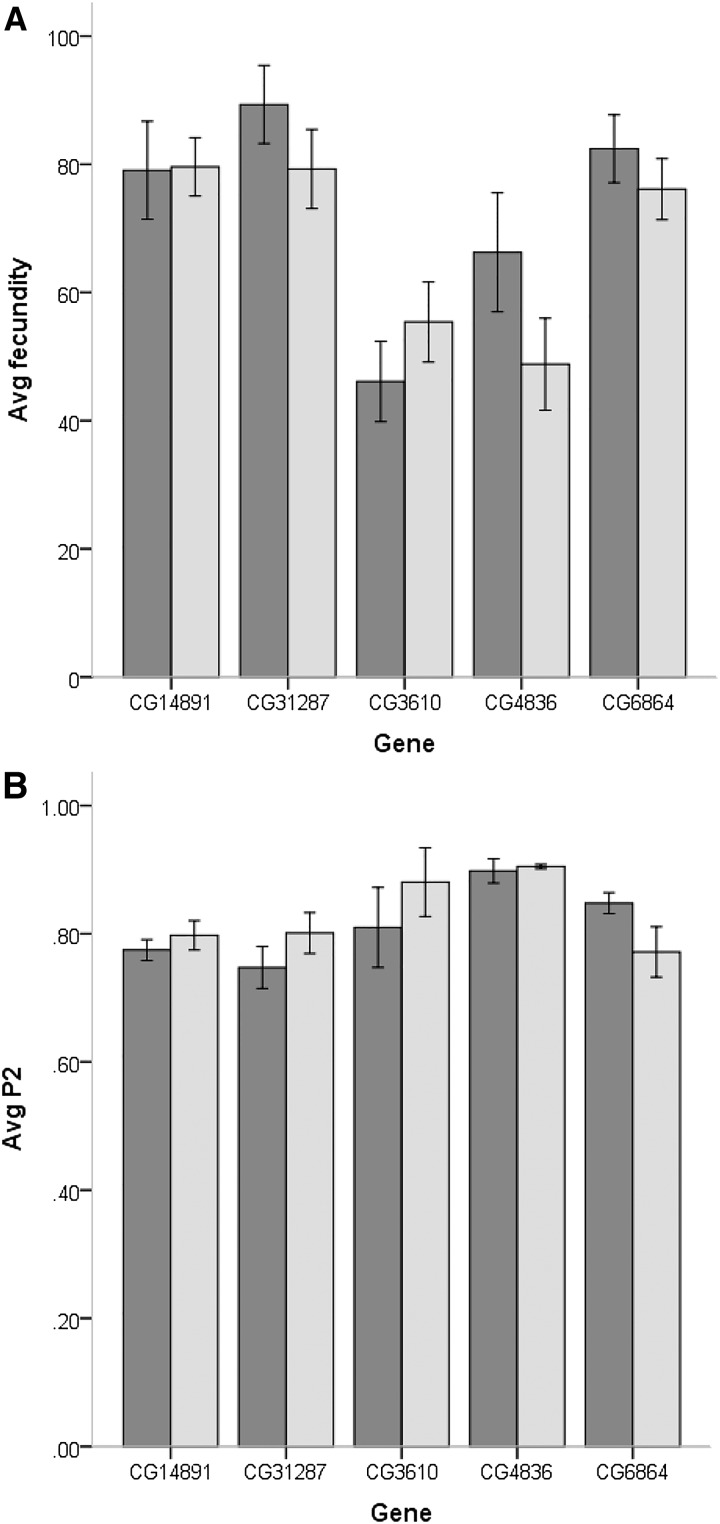
Average fecundity (A) and average second male paternity success (B) of control males (dark gray bar) and gene knockdowns (light gray bars) (A). Error bars represent ± 1 SEM.

**Figure 6:**
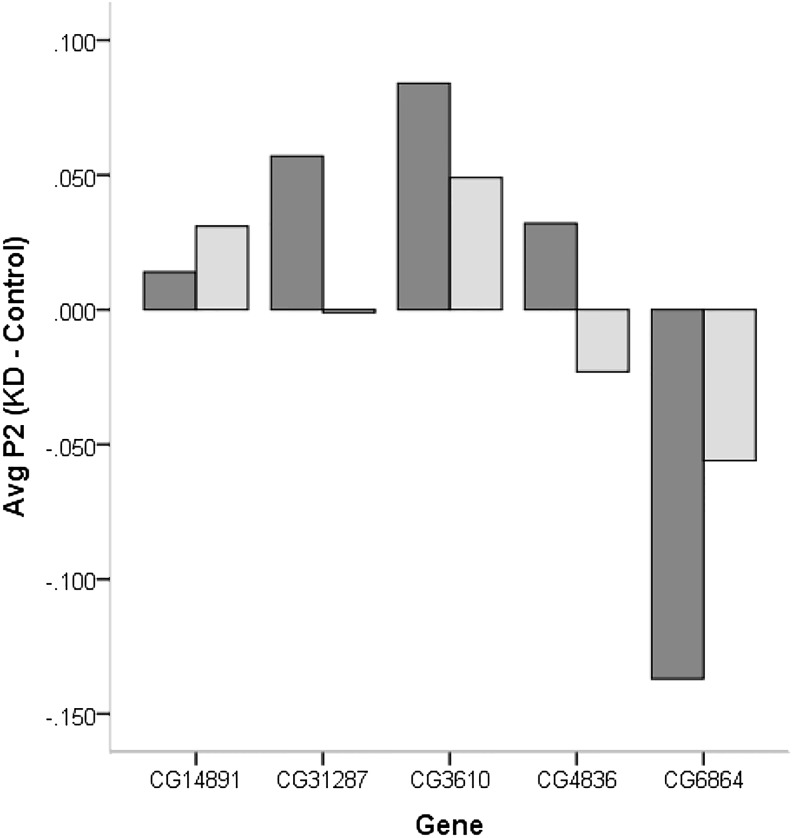
Difference in second male paternity success (P2) between gene knockdown and control males in vial 2 (dark gray bar) and in vial 3 (light gray bar).

We have previously identified eight candidate genes on the basis of mapping differences in CSP using introgression lines and significant differences in male reproductive tract gene expression between genes within the mapped locations. Here, we tested the effectiveness of knockdowns for five (CG6864, CG14891, CG31287, CG3610, and CG4836) of the original eight candidate genes and the effect of the knockdown on second male paternity success (File S2 and File S3). The design allowed us to produce F1 progeny flies with both a Dicer-GAL4 driver and a UAS-RNAi responder that produced a ds-hairpin RNA of the gene to be targeted for knockdown (non-Curly flies) as well as Curly wing flies without the GAL4 driver that were used as controls (Figure 1). Comparisons of gene expression showed a reduction of gene expression in all gene knockdowns. The knockdown for CG4836 showed no significant downregulation compared with Curly flies carrying only the responder (t_6_= 0.80; *P* = 0.231) and CG14891 was only marginally significant (t_7_= 1.77; *P* = 0.083), with all other gene knockdowns showing significant reductions in gene expression relative to controls (Figure 4). None of the knockdowns significantly reduced the average fecundity of males and CG3610 showed an increase, although not significant, in average fecundity of the knockdown males (Figure 5A). Surprisingly, most knockdowns showed a slight, but not significant, increase in second male paternity success, with the effect being the largest for CG3610 (Figure 5B). Only the knockdown of CG6864 (*Mst89B*) had a significantly lower average P2 than the control (t_52_= 1.59; *P* = 0.041) (Figure 5B). The effect was significant immediately after second mating (vial 2; t_51_= 2.12; *P* = 0.014), but not as a delay effect (vial 3; t_49_= 1.12; *P* = 0.115) (Figure 6).

## Discussion

It has been shown in *Drosophila melanogaster* that the amount of sperm stored by the second male to mate and the proportion of progeny he fathers are significantly correlated, suggesting that paternity success of the last male to mate is related to his ability to place sperm in storage and displace resident sperm (Civetta 1999; Manier *et al.* 2010). An actual displacement from the seminal receptacle of resident sperm by incoming sperm had been more accurately described using fluorescent transgenic sperm flies as occurring rather rapidly, with an apparent establishment of an approximately 2-to-1 advantage of the incoming sperm over resident sperm within 72 hr (Manier *et al.* 2010). Given that females spent the first days after second mating in vial 2 before been transferred to vial 3, poor second male paternity success in vial 2 might reflect the incoming sperm inability to properly store and displace resident sperm. The effects of *P*-element gene insert disruptions on sperm storage and displacement ability were only marginal because they reduced average P2 relative to wild-type males but not ebony tester males. Poor second male paternity scores over vial 3 might be linked to deficiencies in sperm physiology and fertilization ability once in storage. Several gene disruptions significantly affected the male’s sperm fertilization ability once in storage. However, some of the fertilization effect (vial 3) could be driven by the marginal effects on sperm storage and displacement (vial 2). For example, males with CG7478 inserts showed significantly lower fertilization ability, but there was an effective increase in their paternity success from vial 2 to vial 3. Similarly, the disruption in fertilization ability of CG31287 was not sufficient to affect the overall second male paternity success of the males. CG6255 gene disruptions did not affect overall second male paternity success, which we attribute to the fact that despite its poor fertilization ability (vial 3 effect) such males did well enough at sperm placement in storage and displacement of resident sperm (vial 2 average P2 = 0.6) to compensate possible deficiencies. Thus, only the disruption of CG14891 affected the overall second male paternity success of its carriers, with the effect of the disruption being particularly strong in vial 3 (fertilization effect). CG14891 is expressed at high levels in the testes but not much is known about its function, except for the suggestion that it might be an F-box protein. F-box genes have gene regulatory roles in a wide variety of functions, including signal transduction, cell proliferation, and growth (Ou *et al.* 2003; Rogers *et al.* 2009).

Most gene knockdowns did not affect fecundity or second male paternity success. We interpret this result in a conservative manner as evidence that such genes do not influence such phenotypes, although there are a few caveats: (1) we cannot rule out that the effect of this single gene knockdown is being rescued by partner genes and (2) the controls we used, although genotypically more equivalent to the knockdown flies than wild-types, were carriers of the hairpin constructs, so they could have been leaky effects even in the absence of the GAL4 driver. The only significant effect of the gene knockdowns was for CG6864 (*Mst89B*) on second male paternity success. The significant effect of this gene knockdown was evident on vial 2, suggesting that the gene defect affects the ability of males to store or effectively displace resident sperm. This result is particularly interesting given that yeast two-hybrid data have suggested interactions between *Mst89B* and *Cdlc2*, a microtubule motor activity protein expressed in the sperm, and *Acp62F*, an accessory gland protein that increases a male’s ability to place sperm in storage (Giot *et al.* 2003; Mueller *et al.* 2008). It is possible to speculate that the interaction among these proteins, and perhaps others, might exert an effect on the sperm ability to reach and properly function in storage. Although the knockdown of *Mst89B* significantly diminished second male paternity, it did not cause a breakdown (*i.e.*, P2 < 0.5) of second male paternity success, indicating a polygenic basis underlying changes in sperm competitive ability and that *Mst89B* might not function as a major gene.

Our results can be interpreted as providing support for the hypothesis that genes can be co-opted from a role in providing an advantage in competitive paternity success within species to underlying the genetics of an interspecies hybridization avoidance phenotype (CSP). The fact that intraspecific mechanisms of sperm competition appear as contributors to CSP (Manier *et al.* 2013c) further supports the possibility of gene co-option. Thus, gene co-option might have facilitated the diversification of a reproductive-related phenotype over a relatively short evolutionary time scale in a manner similar to how it has facilitated the origin of evolutionary novelties during the diversification of other phenotypes in a wide variety of species (Moczek and Rose 2009; Martin *et al.* 2014). However, it is alternatively possible that both genes might have diverged to play roles in CSP as a consequence of pleiotropic effects. Future studies should attempt gene silencing experiments in species other than *D. melanogaster*.

Finally, the localization of both CG14891 and CG6864 (*Mst89B*) in the chromosome region 89B is of interest. Although it is unclear whether the 89B location harbors other potentially important genes, there are some interesting candidates that are localized nearby *Mst89B* (3R:12,092,734.0.12,094,155), such as spermatogenesis genes *asunder* (3R:12,089,993.0.12,092,613) and *gish* (3R:12,098,174.0.12,130,490), as well as *CG14879* (3R:12,171,547.0.12,174,948), a functionally unknown but highly expressed male accessory gland gene (http://flybase.org/). Such genes and others in the regions should be targeted for functional annotation, in relation to male paternity success, in future studies.

## Supplementary Material

Supporting Information

Corrigendum
