# Supplementary material for: Do Candidate Genes Mediating Conspecific Sperm Precedence Affect Sperm Competitive Ability Within Species? A Test Case in Drosophila
Source: G3 (Bethesda). 2014 Jul 16;4(9):1701–7. doi: 10.1534/g3.114.012476 (PMC4169163; doi:10.1534/g3.114.012476)
Supplement: Supporting Information [file supp_4_9_1701__index.html]

Do Candidate Genes Mediating Conspecific Sperm Precedence Affect Sperm Competitive Ability Within Species? A Test Case in Drosophila — Supporting Information 

# Do Candidate Genes Mediating Conspecific Sperm Precedence Affect Sperm Competitive Ability Within Species? A Test Case in *Drosophila*

## Supporting Information for Civetta and Finn, 2014

**Files in this Data Supplement:**

- Supporting Information - Table S1 and Files S1-S3 (PDF, 148 KB)
- Table S1 - List of qRT-PCR primers and their efficiency. (PDF, 49 KB)
- File S1 - Raw data Figure 2 and Figure 3. (.xls, 95 KB)
- File S2 - Raw data for Figure 4. (.xls, 150 KB)
- File S3 - Raw data for Figure 5 and Figure 6. (.xls, 33 KB)
