# Supplementary material for: Do Candidate Genes Mediating Conspecific Sperm Precedence Affect Sperm Competitive Ability Within Species? A Test Case in Drosophila
Source: G3 (Bethesda). 2014 Jul 16;4(9):1701–7. doi: 10.1534/g3.114.012476 (PMC4169163; doi:10.1534/g3.114.012476)
Supplement: Supporting Information [file supp_g3.114.012476_012476SI.pdf]

G3 · Genes | Genomes | Genetics

Do candidate genes mediating conspecific sperm precedence affect sperm competitive ability within species? A test case in *Drosophila*

Alberto Civetta* and Scott Finn

Department of Biology, University of Winnipeg, Winnipeg, MB, R3B 2E9 Canada.

*Corresponding author: E-mail: a.civetta@uwinnipeg.ca

DOI: 10.1534/g3.114.012476

Table S1: List of qRT-PCR primers and their efficiency. All sequences are listed in 5'- 3' direction.

Gene	Primer	Sequence	Efficiency
CG14891	Forward	TCCAACTTGCGGCCCTGTCG	96%
	Reverse	GGAGACGAGACAGACCGCCCA	
CG31287	Forward	GAGGAGGTCGCGACGTATAA	105%
	Reverse	AGCAGCTCGAACTCCTTTTG	
CG6864	Forward	GATCTTCTCCGCAATGCTTC	101%
	Reverse	TGGCATCCAGTGTTTGTCAT	
CG3610	Forward	TCGAATCCCTAGACCAATCG	104%
	Reverse	TATGTGTCAAAGGCACGGAA	
CG4836	Forward	CGATCGCAAGAAGAAAAAGG	102%
	Reverse	GAGTTCTATTGCCCAGCTGC	
Rpl32	Forward	TACAGGCCCAAGARCGTGA	91%
	Reverse	ACCGTTGGGGTTGGTGAG	

Files S1-S3

Available for download as Excel files at http://www.g3journal.org/lookup/suppl/doi:10.1534/g3.114.012476/-/DC1

File S1 Raw data Figure 2 and Figure 3

File S2 Raw data for Figure 4

File S3 Raw data for Figure 5 and Figure 6
