# Supplementary material for: Do Candidate Genes Mediating Conspecific Sperm Precedence Affect Sperm Competitive Ability Within Species? A Test Case in Drosophila
Source: G3 (Bethesda). 2014 Jul 16;4(9):1701–7. doi: 10.1534/g3.114.012476 (PMC4169163; doi:10.1534/g3.114.012476)
Supplement: Supporting Information [file supp_g3.114.012476_TableS1.pdf]

Table S1: List of qRT-PCR primers and their efficiency. All sequences are listed in 5’- 3’ direction.

Gene	Primer	Sequence	Efficiency
CG14891	Forward	TCCAACTTGCGGCCCTGTCG	96%
	Reverse	GGAGACGAGACAGACCGCCCA	
CG31287	Forward	GAGGAGGTCGCGACGTATAA	105%
	Reverse	AGCAGCTCGAACTCCTTTTG	
CG6864	Forward	GATCTTCTCCGCAATGCTTC	101%
	Reverse	TGGCATCCAGTGTTTGTCAT	
CG3610	Forward	TCGAATCCCTAGACCAATCG	104%
	Reverse	TATGTGTCAAAGGCACGGAA	
CG4836	Forward	CGATCGCAAGAAGAAAAAGG	102%
	Reverse	GAGTTCTATTGCCCAGCTGC	
Rpl32	Forward	TACAGGCCCAAGARCGTGA	91%
	Reverse	ACCGTTGGGGTTGGTGAG	
