# Supplementary material for: Do Candidate Genes Mediating Conspecific Sperm Precedence Affect Sperm Competitive Ability Within Species? A Test Case in Drosophila
Source: G3 (Bethesda). 2014 Jul 16;4(9):1701–7. doi: 10.1534/g3.114.012476 (PMC4169163; doi:10.1534/g3.114.012476)
Supplement: Corrigendum [file supp_4_9_1701_v2_index.html]

Corrigendum 

# Do Candidate Genes Mediating Conspecific Sperm Precedence Affect Sperm Competitive Ability Within Species? A Test Case in *Drosophila*

## Corrigendum for Civetta and Finn, G3: Genes|Genomes|Genetics 4 (9) 1701-1707.

**Files in this Data Supplement:**

- Corrigendum
