# Supplementary material for: Do Candidate Genes Mediating Conspecific Sperm Precedence Affect Sperm Competitive Ability Within Species? A Test Case in Drosophila
Source: G3 (Bethesda). 2014 Jul 16;4(9):1701–7. doi: 10.1534/g3.114.012476 (PMC4169163; doi:10.1534/g3.114.012476)
Supplement: Corrigendum [file supp_g3.114.012476_Corrigendum_Civetta.pdf]

Corrigendum for Civetta and Finn, *G3: Genes|Genomes|Genetics* 4 (9) 1701-1707.

G3: Genes|Genomes|Genetics, Vol 4, 1701-1707, September 2014, Copyright © 2014 Genetics Society of America.

CORRIGENDUM

In the article by A. Civetta and S. Finn (*G3* 4:1701-1707) entitled "Do Candidate Genes Mediating Conspecific Sperm Precedence Affect Sperm Competitive Ability Within Species? A Test Case in Drosophila.", in the abstract the annotation ID for Mst89B is indicated as CG6468 rather than CG6864. The abstract has now been corrected in the publication.
